# Combined Exposure to Ecologically Relevant Concentrations of Atrazine and Microcystin Causes Morphological Changes in the Hepatopancreas of Crayfish

**DOI:** 10.1093/icb/icaf012

**Published:** 2025-04-04

**Authors:** Sharita G Reddy, Mariana D Muskovac, Alzhra Alduis, Jada C Manns, Sarah Awali, Antonillamarein Hanna, Luna L Jacob, Patrick Ibrahim, Hasan Alsharifi, Gacia Vosbigian, Hannadi H Chammout, Kenia L Contreras, Reema N Hamdan, Suzanne M Sareini, Dorian K Goolsby, Andrew A Bosah, Evelyn M Rihacek, Kendra R Evans, Rachelle M Belanger

**Affiliations:** Biology Department, University of Detroit Mercy, Detroit, MI 48221, USA; Biology Department, University of Detroit Mercy, Detroit, MI 48221, USA; Department of Chemistry and Biochemistry, University of Detroit Mercy, Detroit, MI 48221, USA; Biology Department, University of Detroit Mercy, Detroit, MI 48221, USA; Biology Department, University of Detroit Mercy, Detroit, MI 48221, USA; Biology Department, University of Detroit Mercy, Detroit, MI 48221, USA; Biology Department, University of Detroit Mercy, Detroit, MI 48221, USA; Biology Department, University of Detroit Mercy, Detroit, MI 48221, USA; Biology Department, University of Detroit Mercy, Detroit, MI 48221, USA; Biology Department, University of Detroit Mercy, Detroit, MI 48221, USA; Biology Department, University of Detroit Mercy, Detroit, MI 48221, USA; Biology Department, University of Detroit Mercy, Detroit, MI 48221, USA; Department of Chemistry and Biochemistry, University of Detroit Mercy, Detroit, MI 48221, USA; Biology Department, University of Detroit Mercy, Detroit, MI 48221, USA; Department of Chemistry and Biochemistry, University of Detroit Mercy, Detroit, MI 48221, USA; Biology Department, University of Detroit Mercy, Detroit, MI 48221, USA; Department of Chemistry and Biochemistry, University of Detroit Mercy, Detroit, MI 48221, USA; Department of Chemistry and Biochemistry, University of Detroit Mercy, Detroit, MI 48221, USA; Department of Chemistry and Biochemistry, University of Detroit Mercy, Detroit, MI 48221, USA; Department of Chemistry and Biochemistry, University of Detroit Mercy, Detroit, MI 48221, USA; Department of Chemistry and Biochemistry, University of Detroit Mercy, Detroit, MI 48221, USA; Biology Department, University of Detroit Mercy, Detroit, MI 48221, USA

## Abstract

Aquatic environments are contaminated through anthropogenic activities, leading to an increase in a variety of pollutants, including pesticides and algal toxins. The cyanobacterium *Microcystis aeruginosa* produces the toxin microcystin with leucine and arginine (MC-LR) and is found in various freshwater environments. MC*-*LR causes liver and tissue damage in aquatic organisms. Atrazine is a commonly applied herbicide in the United States and is toxic following acute exposures. These toxins can often be found together in aquatic environments and thus may lead to combined toxicological effects; however, very little information is available regarding their cumulative effects on tissues such as the hepatopancreas (or liver). To examine the combined effects, we exposed crayfish (*Faxonius virilis*) to 10 ppb atrazine, 10 ppb MC-LR, a combination of 10 ppb of both, and a control for 96 hours. The hepatopancreas was examined and tubular morphology of each group of crayfish was compared. We found that morphological defects such as vacuolization, lumen dilation, and epithelial degeneration were seen following exposures to both atrazine and MC-LR individually and in combination. Combined exposures led to a significant increase in vacuolization of tubular epithelium. Following all exposures, lumen proportion increased, epithelial height decreased, and there was degeneration of the microvillar brush border. Overall, hepatopancreas morphology was significantly altered post-exposure in all treatments. These changes could lead to impairment of hepatopancreas and subsequent changes in biotransformation, detoxification, digestion, reproduction, and molting, causing a reduction in crayfish population size. Furthermore, similar cellular and morphological changes may also occur in other crustaceans inhabiting the same environment.

## Introduction

Aquatic environments are frequently exposed to both natural and anthropogenic stressors. Some of the most common aquatic toxins include herbicides, like atrazine (ATR; 2-chloro-4-ethylamino-6-isopropylamino-1,3,5-triazine), and toxins produced by certain freshwater cyanobacteria. ATR is a broad-spectrum herbicide that has been used in the United States (US) since 1950 and over 300 million kilograms are sold annually. It is heavily applied to crops such as corn, soybeans, and sorghum to control the growth of weeds and to increase crop yields (Kiely et al. 2004; EPA 2013; 2024). ATR enters freshwater environments through run-off, ground water seepage, and evapotranspiration (LeBlanc et al. 1997; Tong and Chen 2002). In freshwater ecosystems, cyanobacteria, like *Microcystis aeruginosa*, may accumulate in large amounts in the summer and fall, forming dense surface scums (or toxic algal blooms) in freshwater environments when nitrogen and phosphorus concentrations and water temperatures are high (Fristachi et al. 2008; Merel et al. 2013; Miller et al. 2017). The cyanobacteria in algal blooms release microcystins (MCs), which are common freshwater cyanobacterial toxins. MC with leucine and arginine (MC-LR) is one of the most prevalent and hazardous types of MCs in freshwater environments (Guedes et al. 2014; Rastogi et al. 2014; Mankiewicz‐Boczek et al. 2015; Buratti et al. 2017). Both ATR and MC-LR are commonly detected xenobiotics in aquatic environments, reaching concentrations above the US Environmental Protection Agency (EPA) allowable limits. Thus, aquatic organisms may be exposed to increased amounts of both chemicals simultaneously.

Freshwater environments containing harmful Microcystis blooms can have concentrations of MCs ranging from 0 to over 3000 ppb (µg L^−1^); however, these concentrations can vary spatiotemporally (Miller et al. 2017). In Lake Erie, MC-LR concentrations up to 14 ppb have been reported (Rinta-Kanto et al. 2009). In 2019, the EPA developed water quality criteria for MCs (limit 8 ppb for swimming); however, environmental concentrations have been found above that limit and it is unknown if these regulations protect the health of aquatic organisms (EPA 2019). The presence of MC-LR in aquatic environments poses issues with water quality management as exposure to MC-LR can alter the physiology of exposed organisms. Physiological changes include compromised antioxidant defenses and a reduction in detoxification enzymes in zebrafish (*Danio rerio*), common carp (*Cyprinus carpio*), and tilapia (*Oreochromis niloticus*), making them more susceptible to environmental stressors and diseases (Prieto et al. 2006; Chang et al. 2020; Chen et al. 2012). MC-LR can be introduced into tissues of aquatic organisms via ingestion of contaminated water through consumption of contaminated food items and may also be absorbed by the gills (Evans 1987; Smith et al. 2008). The half-life of MC-LR can range from 2.2 to 22.2 days depending on temperature and season, therefore MC-LR can accumulate and have a negative impact on aquatic organisms (Ho et al. 2012). Like MC-LR, ATR concentrations also vary widely in the environment. In the US, ATR is extensively applied in the midwestern states in agricultural areas (USGS 2015). Aquatic environments surrounding these areas can received an input of ATR following storm events, and increased ATR concentrations have been detected well above the US EPA limits of 15 ppb. ATR has been detected in some US rivers at concentrations from 0 ppb to well above 300 ppb (EPA 2014; Belanger et al. 2016; EPA 2020). ATR can also be persistent in the aquatic environment as the half-life of ATR can range from six days to several months or years (Comber 1999). ATR enters the body of most aquatic organisms via the gills and is subsequently concentrated in various organs and tissues (Gunkel and Streit 1980). Once absorbed into tissues, both ATR and MC-LR have been found to have wide-spread toxic effects.

The adverse effects of ATR and MC-LR on aquatic organisms are complex and significant and have been widely reported in many aquatic organisms (Solomon et al. 2008; Miller et al. 2017; de Albuquerque et al. 2020; Zhang et al. 2023). Both ATR and MC-LR have been reported as hepatotoxins in many vertebrate and invertebrate species (Li et al. 2005; Zhu et al. 2011b; Santos and Martinez 2012; Galanti et al. 2013; Miller et al. 2017; Chi et al. 2021; Hadeed et al. 2022). As ATR is known to accumulate in the hepatopancreas, it has been shown to induce biochemical changes and DNA damage in several aquatic organisms, including fish (*D. rerio* and *Prochilodus lineatus*) and crayfish (*Faxonius virilis* and *Cherax destrcutor*) (Zhu et al. 2011a; Santos and Martinez 2012; Stara et al. 2018; Awali et al. 2019; Abdulelah et al. 2020; Hadeed et al. 2022). Cyanobacteria harmful algal blooms (cyanoHABs) have increased in size and duration in freshwater habitats (Huisman et al. 2018). Consequently, aquatic organisms will encounter higher concentrations of MC-LR for longer periods of time. Shartau et al. (2022) reviewed several studies that showed that MC-LR affects the liver of many fish species. They noted wide-spread hepatic vacuolization, degeneration, shrinkage, necrosis, and loss of normal liver architecture following MC-LR exposures, with some fish exhibiting liver lesions following only 6 hours of exposure. Further, MC-LR also affects crustaceans as freshwater crabs, crayfish and shrimps, which are known to accumulate MCs in the hepatopancreas (Lirås et al. 1998; Tricarico et al. 2008; Bownik 2013). Crabs (*Chasmagnathus granulata*), Chinese mitten crabs (*Eriocheir sinensis*), and freshwater shrimp (*Palaemonetes argentinus)* also had changes in oxidative stress markers and detoxification enzymes following exposure to MC-LR (Galanti et al. 2013; Sabatini et al. 2015; Chi et al. 2021). Although hepatopancreas MC-LR accumulation and biochemical changes in crustaceans have been noted, morphological changes in the hepatopancreas have not been examined. Further, the joint toxicological effects of cyanobacterial toxins, like MC-LR and commonly found herbicides (e.g., ATR), have not been examined.

Combined effects of toxins can have profound effects on the health of exposed organisms. It should be anticipated that toxic cyanobacteria, like *M. aeruginosa* and therefore MC-LR, can co-occur with a wide range of additional biological and chemical health hazards, including herbicides like ATR (Metcalf and Codd 2020). Microcystins, like MC-LR, and ATR frequently co-occur in freshwater environments, particularly in areas impacted by agricultural runoff and eutrophication. For instance, ATR concentrations in Nebraska watersheds varied from 12 to 125 ppb, with several sites also exhibiting elevated microcystin levels, reaching as high as 230 ppb (NRDC 2007; Loftin et al. 2016). Several studies have examined synergistic effects of MC-LR and other pollutants (e.g., copper, cadmium, phenanthrene, polystyrene microplastics) (Cao et al. 2020; Wei et al. 2020; Wan et al. 2021; Zuo et al. 2021; Ling et al. 2022); however, only one study examined the synergistic effects of ATR and MC-LR (Jiang et al. 2013). Toxic algal blooms can often coincide with other pollutants in the aquatic environmental because surface run-off can be enriched with nutrients and other pollutants, yet the joint toxicological effects of many pollutants on aquatic organisms are still unknown. It has been shown that exposure to other pollutants, like polystyrene nanoplastics and copper, may enhance the toxicity of MC-LR when combined (Wei et al. 2020; Ling et al. 2022). Jiang et al. (2013) exposed fish (*C. carpio*) to both ATR and MC-LR for 14 days and found that there were pathological changes in the liver including ruptured epithelia, apoptosis, pyknosis, and hemolysis. These findings were not compared to treatments of just ATR and MC-LR, so the combined enhancement of toxicity was not investigated.

Crayfish are continuously exposed to their dynamic aquatic environment, where both natural and anthropogenic contaminants can impact their health. However, studies investigating the combined effects of multiple contaminants are limited. While both ATR and MC-LR are commonly found in freshwater environments, little is known about their potential interactions. Jiang et al. (2013) observed significant apoptosis in the hepatic cells of carp (*C. carpio*) after a 14-day exposure to both contaminants. In this study, we aim to explore whether environmentally relevant concentrations of ATR and MC-LR, when combined, produce toxic effects in the hepatopancreas of crayfish (*F. virilis*). We exposed crayfish to 10 ppb of ATR, MC-LR, and a mixture of both to assess potential additive or combined effects. Previous research has shown that individual exposures to MC-LR and ATR caused structural damage to the hepatopancreas of crustaceans, including epithelial degeneration, vacuolization, and abnormal tubule lumens (Chi et al. 2021; Hadeed et al. 2022). When the hepatopancreas of crayfish is damaged, several negative effects can occur, as this organ plays a critical role in digestion, metabolism, and detoxification (Holdich 2002). We hypothesize that simultaneous exposure to both hepatotoxins will lead to more pronounced damage, as seen in the hepatic cells of carp and zebrafish co-exposed to MC-LR and ATR (Jiang et al. 2013). Crayfish were chosen for this study due to their critical role as keystone species and ecosystem engineers (Statzner et al. 2000; Reynolds et al. 2013). Understanding how exposure to these hepatotoxins affects their hepatopancreas is vital, as changes in cellular morphology could impair hepatic function, health, and survival. Moreover, given their ecological importance, any decline in crayfish populations could significantly impact local biodiversity and the broader aquatic ecosystem.

## Methods

### Animals

Male crayfish (*F. virilis*) used in this experiment (weight: 16.9 ± 4.7 g, carapace length: 4.0 ± 0.1 cm, chelae length: 3.0 ± 0.1 cm; mean ± standard deviation, *N* = 16), were obtained from Big Moe’s Bait and Tackle (Detroit, MI, USA) and housed in the aquatic’s facility at the University of Detroit Mercy. They were stored in large tanks for at least two weeks until they were used for this experiment [mean water chemistry parameters: pH 7.5, dissolved oxygen = 8.29 mg L^−1^, dissolved oxygen % saturation = 96.45%, temperature = 22.0°C, hardness = 150 ± 25 mg L^−1^, and TOC = 2.54 mg L^−1^; 14:10 h light–dark cycle]. During this time and throughout the treatments, crayfish were fed one rabbit pellet per crayfish three times per week. Crayfish were divided into four exposure groups (control, ATR-exposed, MC-LR-exposed, and crayfish exposed to both ATR and MC-LR; *N* = 4 crayfish per group). There was no difference in the size of crayfish used for each of the treatment groups tested (one-way ANOVA; F_3,16_ = 2.518; *P* = 0.095). During the experiment, three crayfish (one from each of the control, ATR, and MC-LR treatment groups) likely died due to agonistic interactions in the treatment tanks (Tierney et al. 2000). Following these mortalities, the control, ATR, and MC-LR groups each contained three crayfish, while the ATR and MC-LR treatments contained four crayfish.

### Exposure to atrazine and MC-LR

In this experiment, crayfish were exposed to ATR (10 ppb), MC-LR (10 ppb), and a combination of both ATR (10 ppb) and MC-LR (10 ppb) for 96 hours. This was done by isolating male crayfish in 9.5 L glass aquaria (30 × 15 × 20 cm) containing 5 L of dechlorinated water, air stones, and two polyvinyl chloride tubes (4 cm diameter, 12 cm length) for shelters (*N* = 2 crayfish per treatment tank). ATR (Thermo Scientific; 95% purity) and MC-LR (MilliporeSigma; 95% purity) stock solutions (10 mg L^−1^) were added to dechlorinated water to achieve the final exposure concentrations. Control stock, 0.4% DMSO, was also added to control tanks. To obtain final treatment concentrations, 5 mL of stock ATR or MC-LR solution was added to treatment tanks to obtain the final 4995 mL of dechlorinated water in treatment tanks. For treatments with a combined exposure to both ATR and MC-LR treatments, 5 mL stock ATR and 5 mL stock MC-LR was added to 4990 mL dechlorinated water in treatment tanks. To control for exposure to DMSO in treatment groups, crayfish were exposed to 0.0008% DMSO, final concentration. This was done by adding 10 mL of control stock to 4990 mL of dechlorinated water in exposure tanks. During the 96-h exposure, solutions were refreshed one time, following 48 h of exposure.

All ATR and MC-LR treatment solutions were verified and standardized via liquid chromatography–mass spectrometry (LC–MS) analysis. All reagents were purchased from Sigma-Aldrich (St. Louis, MO, USA). Water, methanol, and acetonitrile were LC–MS grade. ATR and ATR-d_5_ stock solutions of 1.00 mg mL^−1^ each were prepared in methanol. The MC-LR stock solution was prepared by the supplier and was 250 µg per 100 µL of DMSO. Standard solutions of 0, 1, 2, 5, 10, and 50 ppb of ATR and MC-LR were prepared by dilution of stock solutions in water, and 5 ppb ATR-d_5_ was included in all solutions for use as an internal standard for ATR. The standards and exposure solutions were analyzed by reversed phase LC–MS using an Agilent 1290 UHPLC coupled to an Agilent 6220 Accurate-Mass Time-of-Flight Mass Spectrometer (Santa Clara, CA, USA). The column used was a Phenomenex Kinetex 2.6 μm C18 column (2.1 × 75 mm) (Torrance, CA, USA). Mobile phase A was 0.1% formic acid in water, and mobile phase B was 0.1% formic acid in acetonitrile. The LC flow rate was 0.2 mL min^−1^, and the gradient consisted of a 3-min linear ramp from 10 to 90% B, a 1-min wash at 90% B, and a 4-min re-equilibration period at 10% B (total run time 8 min). The injection volume was 20.0 μL, and the column temperature was 30°C. Accurate mass full-scan (m/z 50–1200) detection was performed in positive-ion mode, monitoring for the following ions: ATR, m/z 216.1016; ATR-d_5_, m/z 221.1330; and MC-LR, m/z 995.5566. Other mass spectrometer parameters were as follows: fragmentor voltage 150 V, capillary voltage 3500 V, gas temperature 325°C, gas flow 5 L min^−1^, and nebulizer pressure 30 PSIG. The acquisition time was 1000 ms per spectrum. LC–MS validation of ATR treatment solutions can be found in Table S1 (Supplement).

### Tissue processing and staining

Following treatment, crayfish were placed in a –20°C freezer for 10–15 min before being decapitated. The hepatopancreas was removed by making a ventral incision in the abdomen with scissors, and the hepatopancreas was subsequently weighed. Once the hepatopancreas was isolated, it was cut into 0.5 cm cubes and placed in labeled falcon tubes containing 4% paraformaldehyde at 4°C for at least 48 h. Following fixation, hepatopancreas tissues were prepared for paraffin embedding by treating tissues with 50%, 75%, 95%, and 100% ethanol, 100% xylene and paraffin (15 min; three replicates). The tissues were embedded in a paraffin mold and allowed to cool. The molds were placed in the freezer overnight to fully solidify the paraffin blocks (Bancroft and Stevens 1990). Paraffin-embedded blocks were sectioned (5 μm) using a microtome (Microm HM 325A, Waldorf, Germany) and floated on a water bath. Sections were subsequently collected on microscope slides and dried on a slide warmer before being stained with hematoxylin and eosin (H&E).

Standard H&E protocols were used to determine if ATR and MC-LR exposures led to morphological changes in the hepatopancreas (Bancroft and Stevens 1990; Hadeed et al. 2022). Briefly, sections were deparaffinized with xylene, rehydrated using a descending ethanol series, stained with hematoxylin (5 min), decolorized with acid alcohol, counterstained with eosin (10 min), dehydrated with 95% (1 min; 1 replicate) and 100% ethanol (1 min; 3 replicates), cleared with xylene (1 min; 3 replicates), and mounted with Permount. H&E sections were imaged using a Nikon Eclipse light microscope with a DAGE-MTI colored camera (Tokyo, Japan) an analyzed.

### Analysis and statistics

H&E images were examined, and changes in hepatopancreas pathological features including changes in the epithelium height, vacuolization, and cellular morphology were analyzed and compared for both control and treated crayfish (Desouky et al. 2013; Stara et al. 2018; Hadeed et al. 2022). The examination of the hepatopancreas included measuring the number of vacuoles per tubule from ten representative tubules per crayfish. The total number of tubule cross sections examined per concentration was 30 for control, ATR, and MC-LR treatments (*N* = 10 per crayfish) and 40 for the combined ATR and MC-LR treatment (*N* = 10 per crayfish). Vacuoles that coalesced into one larger vacuole were counted as one vacuole (Xiao et al. 2014; Laurenz et al. 2020). The number of vacuoles per tubule were counted using Cell-Counter, a Fiji plugin (https://imagej.nih.gov/ij/). Fiji was also used to measure the height of the tubular epithelium from the basal surface of the tubule to the apical surface of the epithelium at four equidistant points per tubule and averaging them. Ten random tubule cross sections per crayfish were selected and the proportion of the tubule occupied by the lumen was quantified (Woodburn et al. 2011). Using the Fiji Color Counter plugin (https://imagej.net/ij/plugins/color-counter.html), data for the pixels of the whole tubule and then pixels in just the lumen were obtained. Lumen proportion was calculated for each tubule by dividing the pixels of the lumen by those of the whole tubule for all exposure concentrations (Yu et al. 2018). Proportions were arcsine transformed prior to statistical analysis.

Data for the number of vacuoles per tubule, lumen proportion, and tubular epithelial height were tested for normality. The data for lumen proportion were normal, but the number of vacuoles per tubule and epithelial height were not. Data for the epithelial height were transformed using an orderNorm transformation, and data for the number of vacuoles per tubule were transformed using a Yeo-Johnson transformation (Bartlett 1947; Yeo and Johnson 2000). Given the sample size, a mixed model approach was used, as it accounts for variability both between and within individuals, with multiple cells per N. A linear mixed model method was performed separately using the variables number of vacuoles per tubule, epithelial height and lumen proportion with treatment as a factor and animal ID as a random factor (Schielzeth and Nakagawa 2013; Brown 2021) using the lme4 package in R statistical software (Bates et al. 2015; R Development Core Team 2019). Animal ID was needed as a random factor given multiple measures taken from the same animal. An emmeans post hoc with a Tukey-HSD adjustment was used to compare the different treatments and the ATR, MC-LR, and ATR and MC-LR treatments (Lenth 2024).

## Results

**Fig. 1:**
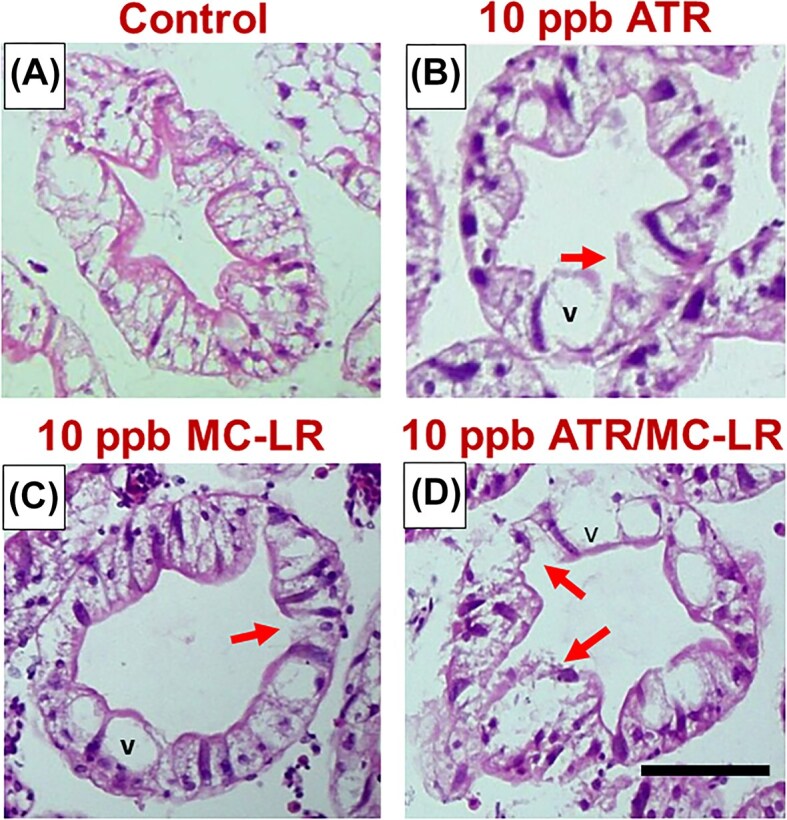
Representative transverse H&E-stained sections of tubules from the hepatopancreas of control (A), ATR-treated (B), MC-LR treated (C), and crayfish treated with both ATR and MC-LR. When crayfish were treated with ATR and MCLR, there were increases in lumen diameter and the characteristic asterisk shape was lost. Secretory (blister-like) cells containing vacuoles (v), known to occur following exposure to xenobiotics, were also noted post-exposure. Moreover, epithelial degradation and necrosis of the microvillar brush border was noted in many tubules following exposure to ATR and MC-LR (see arrows). Scale bar is 50 µm.

H&E staining was used to examine the hepatopancreas tubules of control crayfish and those exposed to environmentally relevant concentrations of ATR, MC-LR, and a combination of both (Fig. 1). Following acute exposures, there were changes noted in the morphology of tubules of the hepatopancreas. In control sections, characteristic asterisk-shaped lumens were visualized, and the microvillar brush border was unbroken and uniform in all sections examined. Small lipid vacuoles were also noted with very few B cells containing large vacuoles (Fig. 1A). An increase in the presence of vacuoles was noted following exposure to ATR and MC-LR. Further, the tubular lumens appeared dilated and exhibited degenerated epithelium with necrosis of microvilli (Fig. 1 B–D).

**Fig. 2:**
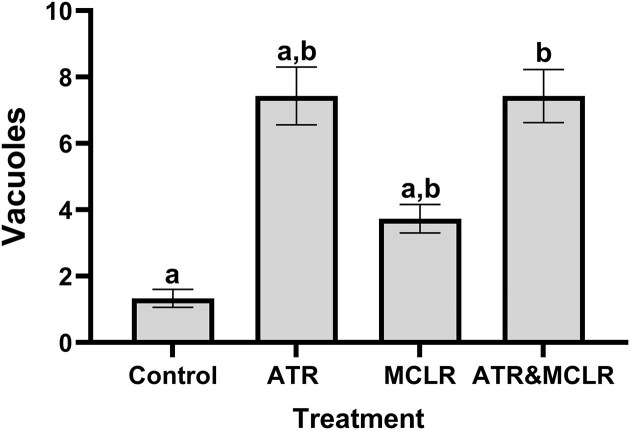
Hepatopancreas tubules of crayfish that were exposed to both ATR and MC-LR had significantly more vacuoles (mean ± S.E.) following the combined ATR and MC-LR exposure when compared to control crayfish. There was no significant difference between control and ATR only and MC-LR only treatments. Treatment groups are assigned different letters (a, b) when they were significantly different (*P* < 0.05).

Following exposures to ATR, MC-LR, and a combination of both, the number of B cells containing large vacuoles increased [F_(3,8,0.05)_ = 4.814; *P* = 0.033; Fig. 2]. Control crayfish, exposed only to DMSO, had 1.33 ± 0.27 [mean ± standard error (S.E.)] vacuoles per tubule. Following exposure to a combination of ATR and MC-LR, there was a significant increase in vacuoles per tubule, compared to the control. Crayfish that were exposed to a combined ATR and MC-LR had 7.43 ± 0.80 vacuoles per tubule, which was significantly more than the control crayfish (*P* = 0.040). When compared to the control group, there was no difference in the number of vacuoles per tubule in the ATR only and MC-LR only treatment groups. They had 7.43 ± 0.87 (*P* = 0.053) and 3.73 ± 0.43 vacuoles per tubule (*P* = 0.355), respectively, which was not significantly different from the control crayfish (Fig. 2).

**Fig. 3:**
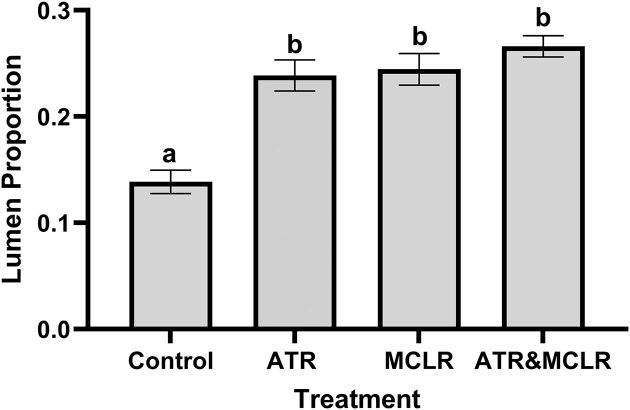
The proportion of the tubule occupied by the lumen (mean ± S.E.) was calculated for each of the treatments (control, ATR, MC-LR, and ATR and MC-LR combined) using Fiji. The tubule lumens of crayfish exposed to 10 ppb ATR, 10 ppb MC-LR, and a combination of 10ppb ATR and 10 ppb MC-LR were significantly increased compared to control; however, they were not different from each other. Control and treatment groups were assigned different letters (a, b) when they are significantly different (*P* < 0.05).

**Fig. 4:**
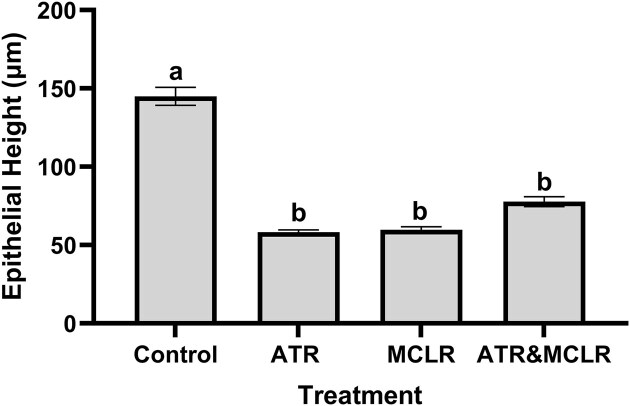
Epithelium height (mean ± S.E.) was significantly reduced following exposure to ATR, MC-LR, and a combination of both ATR and MC-LR. Significant epithelial atrophy and degeneration occurred following exposures to ATR, MC-LR and both ATR and MC-LR. Different letters (a, b) indicate a there was a significant reduction in epithelial height (*P* < 0.05).

The proportion of the tubule occupied by the lumen was calculated using Fiji and compared. There was a significant difference across treatments when lumen proportion was compared [F_(3,8,0.05)_ = 18.03, *P* = 0.0006; Fig. 3]. The lumen proportion for the control group was 0.14 ± 0.01 while the lumen portion was significantly larger for all treatment groups when compared to control treatments (*P* = 0.003, *P* = 0.002, and *P* = 0.001 for crayfish exposed to ATR only, MC-LR only, and a combination of both ATR and MC-LR, respectively). For treatments that only received ATR or MC-LR, the lumen proportion was 0.24 ± 0.01 for both treatments. Following exposure to a combination of both ATR and MC-LR, the lumen proportion was 0.27 ± 0.01. As lumen proportion increased, epithelial height decreased significantly for all treatments [F_(3,8,0.05)_ = 20.226, *P* = 0.0004; Fig. 4]. Tubular epithelium height for control crayfish was 144.96 ± 5.76 µm. Following ATR exposure, this height significantly (*P* = 0.001) decreased to 58.17 ± 1.59 µm. There was also a significant decrease (*P* = 0.001 and 0.014) for MC-LR only and ATR and MC-LR treatments. The epithelial heights were 59.68 ± 2.02 µm and 77.69 ± 3.16 µm, respectively. Overall, it is important to recognize that the results and interpretation of these findings should be thoughtfully considered, as the addition of one or two more crayfish could affect the outcomes. Therefore, the conclusions drawn should acknowledge that a larger sample size may lead to more robust results.

## Discussion

ATR and MC-LR are prevalent hepatotoxins of anthropogenic and natural origins, yet studies on their combined effects remain limited. In this study, crayfish were acutely exposed to ATR, MC-LR, and their combination and hepatopancreas morphology was examined. Observed morphological changes included increased vacuolization, epithelial degeneration, disintegration of the microvillar brush border, and dilation of the tubular lumen. These changes suggest that combined exposures exacerbate the toxicological effects of ATR and MC-LR compared to individual exposures. We acknowledge that the results of dual-factor experiments, like this one, with small sample sizes, may be affected by the addition of even one or two more crayfish (McNeish and Stapleton 2016). Consequently, the results could change as a larger sample sizes could alter the study’s outcomes. Morphological changes to the hepatopancreas of crayfish can serve as indicators of physiological disturbances and can therefore be utilized to assess environmental quality (Antón et al. 2000; Holdich 2002; Belanger et al. 2017; Awali et al. 2019; Hadeed et al. 2022). Moreover, given the importance of the hepatopancreas for growth, molting, and biotransformation of xenobiotics into inactive forms, changes in the physical, physiological, and morphological state of this organ, like the ones noted in this study, may result in changes in the overall health of the animal (Holdich 2002; Belanger et al. 2017).

Vacuoles are enzyme storing compartments, and increases in these vacuoles are known to occur following exposure to xenobiotics, including pesticides and natural toxins (Vogt 1987; Antón et al. 2000; Vogt 2019; Chi et al. 2021; Hadeed et al. 2022). Vacuole formation, associated with the uptake, biotransformation, and storage of xenobiotics (Icely and Nott 1992; Ning et al. 2020), increased significantly following combined ATR and MC-LR exposure, though individual exposures had lesser effects (Figs. 1 and 2). Similar vacuolization and hepatopancreatic damage have been reported in studies involving pesticides, heavy metals, and cyanotoxins, emphasizing the hepatopancreas’s sensitivity to environmental stressors (Jiang et al. 2013; Abdulelah et al. 2020; Chi et al. 2021). When carp were also exposed to a combination of 5 ppb ATR and MC-LR, an increase in hepatocyte vacuolization was also observed in their liver (Jiang et al. 2013). Other pollutants, including the metals zinc, mercury, and cadmium, and pesticides glyphosate and chlorpyrifos, also led to increases in the number of vacuoles in the hepatopancreas in crustaceans (Wu et al. 2008; Yamuna et al. 2009; Liu et al. 2013; Negro and Collins 2017; de Melo et al. 2019). Due to their importance for transformation and removal of xenobiotics, the presence of vacuoles can be used as an indicator to assess sublethal toxicity of a single contaminant or a mixture.

Functional impairments of physiological processes of the hepatopancreas often correspond with changes in morphology. In this study, we found that exposure to ATR and MC-LR led to decreased epithelial height and lumen dilation of the tubules (Figs. 3 and 4). These morphological changes have been linked to disruption in nutrient absorption and detoxification processes (Speck and Urich 1970; Awali et al. 2019; Stara et al. 2018; Steele et al. 2018). Exposure to contaminants, like ATR and MC-LR, leads to cellular necrosis of this epithelium, visible cellular changes, and finally to organ dysfunction. This dysfunction was seen following exposure to both ATR and MC-LR. Crayfish (*F. virilis* and *C. destructor*) had changes in their biochemical profile following ATR exposures (Stara et al. 2018; Awali et al. 2019; Hadeed et al. 2022). Antioxidant biomarkers [e.g., superoxide dismutase, catalase, glutathione reductase, glutathione S-transferase (GST), reduced glutathione] and detoxification enzymes, cytochrome P_450_ and GST, were altered following exposures. These biochemical changes correlated with alterations in hepatopancreas morphology. Chi et al. (2021) found that in addition to morphological changes visualized in the hepatopancreas of Chinese mitten crabs, MC-LR exposure led to increases in reactive oxygen species levels and altered antioxidant systems (e.g., superoxide dismutase, GST, glutathione peroxidase, glutathione reductase activities, and glutathione content). Given the importance of the hepatopancreas for biotransformation and detoxification, any degenerative changes will lead to changes in the ability to remove harmful substances from the body.

Our findings underscore the potential long-term consequences of ATR and MC-LR exposures on crayfish populations. Mixtures of toxins can have distinct effects at different concentrations and lengths of exposure. For example, additive effects of MC-LR and phenanthrene were only experienced at higher concentrations (Wan et al. 2021). Further, toxic effects were also noted when *Daphnia magna* were chronically exposed to lower concentrations of MC-LR and phenanthrene. These results suggest that exposure to lower concentrations of MC-LR and ATR could have additive toxic effects following a chronic exposure. Naturally, both ATR and MC-LR concentrations, as well as exposure durations, fluctuate. ATR has been found at concentrations ranging from 0 to > 300 μg L^−1^ for up to 21 days (EPA 2014). MC-LR can be found at concentrations ranging from less than 1 μg L^−1^ to over 2 mg L^−1^, and these higher concentrations of MC-LR can be found for over three weeks in some aquatic environments (Lahti et al. 1997; Orihel et al. 2012). Collectively, a chronic exposure to both ATR and MC-LR may have detrimental effects on hepatopancreas morphology and function. Overall, altered hepatopancreas morphology and function may impair growth, molting, and survival, ultimately reducing population sizes.

## Conclusions

Significant morphological changes including irregularly shaped and dilated tubule lumens, microvillar brush border disintegration, and epithelial tissue degeneration were observed post exposure to ATR and MC-LR. Additionally, there was an increase in lumen size in the hepatopancreas tubules and an increase in B-cell vacuolization. Collectively, this indicates that exposure to both natural and anthropogenic toxins affect the hepatopancreas. Changes in morphology may be correlated with changes in function and overall health as Awali et al. (2019) previously showed that following ATR exposures, there were changes in the expression and activity of the detoxification enzymes cytochrome P_450_ and GST. Further, reallocating energy resources for cell and tissue repair as well as biotransformation and detoxification of ATR, MC-LR and other xenobiotics may be detrimental to the long-term health of the animal. Overall, because the hepatopancreas serves as the main energy reserve for growth and molting and is the main organ for detoxification, any impairments in this organ could lead to decreased chance of survival and ultimately affect population size. Management strategies must address these combined stressors to protect aquatic ecosystems from pollutants. Overall, comprehensive studies on the interactions of cyanotoxins and other xenobiotics are essential for informed regulatory decisions and are critical to protecting aquatic organisms and their ecosystems.

## Supplementary Material

icaf012_Supplemental_Files

## Data Availability

Data are available in a repository and can be accessed via https://doi.org/10.7910/DVN/F80XSV.
