# Supplementary material for: Combined Exposure to Ecologically Relevant Concentrations of Atrazine and Microcystin Causes Morphological Changes in the Hepatopancreas of Crayfish
Source: Integr Comp Biol. 2025 Apr 4;65(1):168–77. doi: 10.1093/icb/icaf012 (PMC12284762; doi:10.1093/icb/icaf012)
Supplement: icaf012_Supplemental_Files [file icaf012_supplemental_files.zip › Table S1.docx]

Supplement:

**Table S1.** RPLC-MS was used to verify the of stock and treatment concentrations of control, ATR, and MC-LR for treatments performed in this experiment. The recorded stock and treatment concentrations were consistent with those selected for the experiment.

| **Stock and Treatment Solutions** | **Mean [ATR] ± S.D. (ppb)** | **Mean [MC-LR] ± S.D. (ppb)** |
| --- | --- | --- |
| Control Stock | 0 | 0 |
| ATR Stock | 10,400 ± 100 | 0 |
| MC-LR Stock | 0 | 9,380 |
| Control Treatment | 0 | 0 |
| ATR Treatment | 9.66 ± 0.94 | 0 |
| MC-LR Treatment | 0 | 9.30 ± 0.65 |
| ATR and MC-LR Treatment | 10.3 ± 0.6 | 9.81 ± 0.57 |
