# Supplementary figures and images for: Combined Exposure to Ecologically Relevant Concentrations of Atrazine and Microcystin Causes Morphological Changes in the Hepatopancreas of Crayfish
Source: Integr Comp Biol. 2025 Apr 4;65(1):168–77. doi: 10.1093/icb/icaf012 (PMC12284762; doi:10.1093/icb/icaf012)

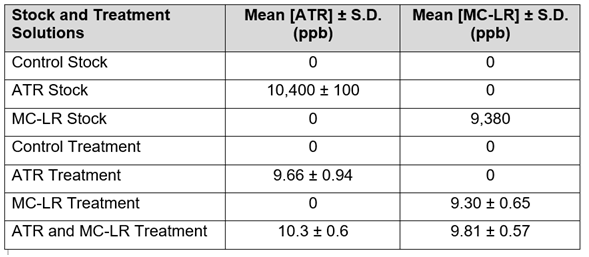

Supplement: icaf012_Supplemental_Files [file icaf012_supplemental_files.zip › Table S1.tif]
